# A Longitudinal Analysis of the Relationship Between Self-Determined Motivation and Prosocial Orientation of Volunteer Tourists: The Mediating Role of Identity Processing Styles in Young Adults

**DOI:** 10.3390/bs15050588

**Published:** 2025-04-27

**Authors:** Liyuan Zhang, Anmin Huang

**Affiliations:** College of Tourism, Huaqiao University, Quanzhou 362021, China; zlylyxy@stu.hqu.edu.cn

**Keywords:** autonomy, egoism, altruism, identity styles, volunteer tourism

## Abstract

The motivational dimensions of volunteer tourists have been a focal point of scholarly debate, closely aligning with their prosocial orientations. Limited attention has been given to examining these relationships through the lens of identity formation among young tourists. To address this gap, this study conducted a two-wave longitudinal investigation into the linkage between volunteer tourists’ self-determined motivation and their prosocial orientation, with identity processing styles serving as a mediator. The sample (*n* = 291) consisted of freshmen and sophomores participating in a volunteer teaching program during the summer of 2024. We measured the variables of interest both before and after their attendance. In addition to direct effects, our findings observed two significant indirect pathways: (1) Wave 1 autonomous motivation was positively associated with an informational identity style at Wave 1, which predicted Wave 2 other-oriented helping tendency; and (2) Wave 1 controlled motivation was positively related to a normative identity style at Wave 1, which accounted for self-oriented helping propensity at Wave 2. Furthermore, the first relationship was uni-directional, as the coefficient for the reverse pathway (i.e., Wave 1 other-oriented helping → Wave 1 informational identity style → Wave 2 autonomous motivation) was not significant. In contrast, the second relationship was bi-directional, wherein Wave 1 self-oriented helping was positively associated with a normative identity style at Wave 1, which subsequently predicted Wave 2 controlled motivation. Taken together, our study contributes to the literature on altruistic motives, prosocial behavior, identity development, and volunteer tourism.

## 1. Introduction

Volunteer tourism, also known as voluntourism, represents an alternative form of travel where tourists are organized to engage in community-based projects for social, environmental, educational, or scientific purposes (Wearing, 2001). With the aim of generating positive impacts on the community well-being of the local destination, i.e., “making a difference”, this niche tourism provides a transformative opportunity for participants to venture beyond their comfort zones to cultivate self-discovery and self-development. Owing to its dual benefits for personal growth (e.g., empathy and understanding enhancement, skill acquisition, career opportunities) and community development (e.g., cultural exchange, environmental conservation, economic stimulation), volunteer tourism has seen rising popularity in recent decades and become increasingly prominent in both media coverage and academic research. As reported by The Guardian (Godin, 2021), each year, over 10 million international travelers, primarily from North America and Europe, go abroad to engage in voluntary work in underprivileged and impoverished countries. In the tourism literature, a heated debate has arisen surrounding the unique incentives of volunteer tourists, with a particular focus on the extent to which they are motivated by self-interest or altruism (Wearing & McGehee, 2013). To address this issue, Callanan and Thomas (2005), categorized volunteer tourists into three groups: shallow, intermediate, and deep. They then explained that shallow volunteers are predominantly driven by personal interests, whereas those falling under the “deep” category tend to prioritize their considerations for the destination community. Vastly different from Callanan and Thomas’s (2005) topology, Mustonen (2007) tended to argue that tourists originally seeking a vacation experience might transform into genuine volunteers through meaningful interactions with both fellow tourists and hosts, even if their initial motives were ego-centric. As such, the motivational aspect of volunteer tourism can be dynamic and prone to being reshaped by the actual experiences encountered during the journey. Tourism researchers have also conceptualized voluntourism as a hybrid business model (Kabil et al., 2023), in which economic viability intersects with ethical imperatives. This commercial–social duality directly shapes how participants interpret their roles, engage in identity processing, and derive motivational meaning from the experience.

Interestingly, the debate over volunteer tourists’ motivations resonates naturally with a dichotomy in prosocial helping, which distinguishes between self-oriented and other-oriented subtypes (Coghlan, 2015). Self-oriented helpers are energized by individual motives and benefits, striving to satisfy one’s own needs or gain personal advantages. In contrast, other-oriented helping is typical of undertakings that prioritize the welfare of and support for others, inspired by empathy, altruism, or social responsibility. This dichotomization of prosocial helping has shed light on meaningful social interactions and the dynamics of social relationships within communities (Stoeber, 2015). At the same time, the self-determination theory (Deci & Ryan, 1985) is also relevant to the “self-interest versus altruism” debate and can be instrumental for conceptualizing motivations that drive volunteer tourism, as it differentiates between intrinsically and extrinsically motivated volunteers. Intrinsic motivation refers to engagement in an activity purely due to an inherent interest or personal self-realization, such as a genuine willingness to make contributions to the well-being of others and society. Conversely, extrinsic motivation is mainly triggered by external factors, such as pursuing interpersonal recognition and fulfilling societal expectations, as well as obtaining tangible rewards. SDT presumes that intrinsic motivation, characterized by greater autonomy and alignment with one’s personal values, is more likely to foster sustained engagement, deeper psychological well-being, and long-term commitment. Conversely, extrinsic motivation, while facilitating the initial participation, pales in being conducive to enduring involvement or transformative experiences. Prior studies have independently substantiated the correlation between autonomy, a core tenet of intrinsic motivation, and the propensity for other-oriented helping (Roth, 2008), as well as the association between personal considerations, such as reputational benefits and favorable self-evaluations, and the tendency for self-interested helping (Spitzmuller & Van Dyne, 2013). However, there remains a dearth of research that explicates the link between self-determined motivation and prosocial orientation among volunteer tourists in a single study.

Another noteworthy trend in the literature regarding the motivational reasons for volunteer tourism is its disproportionate focus on youth volunteers. This particular emphasis can be reasonably attributed to three key factors: the majority of volunteer tourists are young individuals, with over 65% of them being enrolled college students, recent graduates, or those about to enter college, not to mention those who are attending international ”gap year” travel programs; the active participation of youths in volunteer tourism has been demonstrated to exert significant influence on both their internal and external transformations; and volunteer tourism produces long-term effects on young travelers by fostering a sustained commitment to social and environmental causes over a lifetime. Furthermore, considering the essential developmental stage of youth in identity formation and transformation, there has been a growing body of literature that highlights the transformative role of volunteer tourism experiences in shaping visitors’ contemporary identity and its broader influences beyond engagement per se (Butcher & Smith, 2010; Magrizos et al., 2021). It is also noted that young volunteer tourists are very likely to confront a “shifting identity” dilemma, finding it difficult to reconcile their contrasting roles as leisure tourists and volunteer tourists (Coghlan, 2015). This challenge may ultimately lead up to severe tension and incongruency in self-identification during their travel experiences, wherein role ambiguity functions as a major hindrance to the realization of the expectations and promises embedded in volunteer tourism.

Extant research has not fully addressed the motivational dimension of volunteer tourism, as it primarily concentrates on experiences during or after the volunteering period, rather than prior to it or throughout. Therefore, little is known about what systematic social–cognitive strategies young volunteers employ in identity formation related to their tourism motivation, be it self-interested or altruistic. Previous research has pointed out that identity processing styles can generate differential effects on the development of self- and other-oriented helping behaviors (Smits et al., 2011). For example, individuals adopting an information-oriented style, which is characterized by autonomous and open-minded exploration of identity options, would exhibit a stronger inclination towards other-oriented helping. On the other hand, a normative style, which is typified by controlled functioning and a narrow-minded attitude, was predicted to correlate mainly with self-oriented helping behavior. With this in mind, the current study aims to capitalize on young volunteers’ identity processing style as a mediator to longitudinally investigate the relationship between their self-determined motivation and prosocial orientation. We focused on Chinese college students who attend volunteer teaching programs, accounting for a substantial young demographic. By the end of 2018, China had over 50 million registered volunteers in education-related initiatives. Of these, more than 270,000 college students volunteered in northwest and southwest China, making significant contributions to local education, healthcare, agricultural technology, and poverty alleviation campaigns.

## 2. Literature Review

### 2.1. Motivations for Volunteer Tourism

Volunteer tourists tend to seek a distinctive travel experience that diverges vastly from mainstream tourism expectations (Andereck et al., 2012). The motivational dimension of volunteer tourism extends beyond personal development and recreation purposes to interpersonal, communal, environmental, and humanitarian considerations, including cultural communication and exchange, positive contribution to the host community, ecological restoration and conservation, and medical aid service (Raymond & Hall, 2008; Zahra & McGehee, 2013). In the literature, accordingly, there has been discussion about pre-trip motivations of volunteer tourists on a continuum between self-interest and altruism (Wearing & McGehee, 2013). Some factor analytical results support a binary classification by indicating that egoistic motives are pronounced in explaining young volunteers’ in-role behaviors, whereas altruistic concerns serve as a predictor for their extra-role behaviors and evaluation of satisfaction (Cornelis et al., 2013). Moreover, the broader literature on volunteering is inclined to conceptualizing it as organized help, which is thoughtfully mapped out in lieu of a spontaneous decision (Güntert et al., 2016). Likewise, social psychologists often capitalize on a utilitarian approach to rationalize volunteerism wherein individuals may adopt similar attitudes or engage in comparable behaviors with the intention of achieving a particular end, even if these attitudes or behaviors serve drastically different psychological functions (Snyder et al., 2000).

Additionally, researchers have investigated the motivational reasons for volunteer tourism through the lens of SDT, qualitatively dichotomizing a tourist’s motivation as either intrinsically determined or extrinsically controlled (Ryan & Deci, 2000). When individuals are incentivized primarily by intrinsic motivation to engage in a particular activity, they tend to perceive it as inherently challenging, interesting, and enjoyable. Conversely, people are likely to engage the extrinsic side of motivation when they participate mostly for the sake of obtaining external rewards or benefits separable from the activity per se. SDT presumes that the two distinct types of motivation can be distinguished by the extent to which the revealed behaviors become integrated, internalized, or absorbed into the self (Ryan & Connell, 1989). Drawn on the SDT framework, Güntert et al. (2016) demonstrated that the functional aspects of volunteer tourism can be systematically differentiated based on the weight attached to the self-determined or controlled motivation, which in turn influences the level of satisfaction. SDT also underscores that individuals’ personal development and psychological well-being depend on how well their fundamental needs for autonomy, competence, and relatedness are addressed and fulfilled (Ryan & Deci, 2000). In a similar vein, Bidee et al. (2013) identified a positive correlation between volunteer tourists’ exerted efforts and their autonomous motivation, i.e., the freedom to choose actions in alignment with their own personal values and identity. The business model perspective of voluntourism also sheds light on how SDT should be interpreted within this context. Highly commercialized voluntourism programs may inadvertently frustrate the core psychological needs outlined in SDT (Deci & Ryan, 2000). That is, rigid, pre-structured itineraries can constrain autonomy; superficial or symbolic tasks can limit opportunities for developing competence; and minimal or staged interaction with local communities can diminish the sense of relatedness essential for sustaining intrinsic motivation (Sin, 2009; Kabil et al., 2023).

In addition to anatomizing motivations for volunteer tourism from theoretical perspectives, existing research has empirically considered demographic characteristics such as age. Researchers observed that the younger group of volunteer tourists had a higher chance of self-reporting egoistic motivation (Lepp, 2008; Wearing et al., 2008), probably owing to the fact that they were currently experiencing a transitional phase from K-12 education to college or a professional career (Simpson, 2004). On the other hand, middle-aged volunteer tourists were largely driven by a desire for interpersonal connection through constructing relational bonds with the host community, whereas the oldest group, though not fully cognizant of their motivations, frequently leaned towards altruistic impetus in their engagement with volunteer tourism (McGehee & Andereck, 2009). That being said, cross-age studies also revealed that a commonly shared motivation among volunteer tourists was to quest for novel experiences and provide tangible assistance and support to those underprivileged others (Carter, 2008; Stoddart & Rogerson, 2004). Considering that our study aims to examine the dynamic and potentially transformative interplay between volunteer tourists’ self-determined motivation and their prosocial orientation, we focused on the young adult age group, particularly freshmen and sophomores.

### 2.2. Prosocial Orientation

Prosocial orientation refers to an individual’s predisposition to engage in behaviors that are beneficial to others, typically driven by inherent motives such as empathy, altruism, or a sense of moral obligation (Eisenberg et al., 2014). Researchers have long been interested in examining how prosocial behaviors (e.g., helping, sharing, and cooperating) contribute to both positive personal and societal outcomes. A plethora of research indicates that individuals with a strong prosocial orientation are more likely to sustain positive interpersonal relationships, accumulate substantial social capital, and experience greater life satisfaction (Espinosa et al., 2022; Helliwell et al., 2017). In addition, prosocial behaviors have been demonstrated to help improve mental and emotional well-being, fostering senses of fulfillment, gratitude, or stress relief that arise from helping others (Miles et al., 2022). While prosocial conducts are often deemed spontaneous and voluntary, many studies suggest that social and cultural factors significantly impact one’s engagement in these actions. For instance, collectivist cultures structurally prioritize communal welfare and cultivate a more intense atmosphere that encourages prosocial behaviors compared to individualistic cultures (Feygina & Henry, 2015).

In the literature, prosocial behavior is frequently dichotomized into self-oriented and other-oriented forms. Batson (1991) distinguished between egoism (helping to benefit oneself) and altruism (helping for the benefit of others) while investigating the psychological mechanisms of empathy and distress behind both. Likewise, Schroeder et al. (1995) reviewed theories and psychological mechanisms underlying prosocial behavior, touching upon both emotional and cognitive factors that influence helping. Their summary revealed that self-interested helping is often incentivized by the desire to alleviate one’s own negative emotional states, benefits of social exchange, or the anticipation of future reciprocity, whereas other-oriented helping can be explained by a sense of moral responsibility or genuine concern for others’ well-being. In addition to conceptual research, empirical studies have examined sociopsychological factors such as organizational fairness and parental practices in shaping these two polarized prosocial orientations. Bobocel (2013) observed that employees’ responses to unfair events, in the form of forgiveness or revenge, are influenced by their perceptions of organizational justice and whether they have a self- or other-oriented focus, with other-oriented individuals more likely to forgive and self-oriented individuals more likely to suppress revenge when perceiving the organization as fair. It was found in the work of Roth (2008) that parental conditional regard is positively associated with introjection internalization and self-interested orientation, while autonomy-supportive parenting predict fuller internalization and other-oriented helping.

Within the context of volunteer tourism, self-oriented prosocial behavior typically involves individuals participating in volunteer activities for the sake of self-benefit, including seeking personal fulfillment, obtaining social recognition, and, ultimately in a more tangible sense, boosting their resumes (Foller-Carroll & Charlebois, 2016). Volunteers motivated by self-interest tend to pay disproportionate attention to how the experience helps them grow or provides them with opportunities for networking or personal development. Regardless of outwardly prosocial actions, their endogenous incentives are essentially centered on their own needs or goals. On the contrary, other-oriented prosocial behavior in volunteer tourism occurs when individuals are primarily motivated by a genuine desire to benefit others (Mustonen, 2007). Thus, volunteers with an other-oriented focus are wholeheartedly committed to improving the lives of the communities they visit and serve, prioritizing the welfare of others and contributing to social change without expecting personal rewards or social recognition. The balance between self- and other-oriented motivations in volunteer tourism can vary, with some volunteers exhibiting a mix of both, whereas others may lean more towards one orientation (Coghlan, 2015). However, research indicates that other-oriented motivations outcompete self-oriented ones in leading to meaningful and sustainable impacts for both volunteers and communities, as the other-oriented volunteers have a higher intention to passionately and devotedly invest time and efforts in the long-term development of the project or cause (Coghlan & Fennell, 2009). Prosocial orientation functions both as an antecedent and an outcome in the voluntourism experience. Individuals with strong prosocial values are more likely to engage in voluntourism, while elaborately designed programs can further cultivate these values. The underlying business model critically shapes this dynamic. That is, authentic, community-centered initiatives tend to reinforce prosocial tendencies, whereas highly commercialized or performative models may erode them.

### 2.3. Identity Processing Style

Identity processing style denotes an individual’s preferential differences in the social–cognitive strategies employed to either embrace or avoid the undertakings related to identity construction and maintenance. Berzonsky (1988, 1990) developed a model that classifies three distinct identity processing styles: informational, normative, and diffuse–avoidant. The informational style is characterized by proactively seeking, assessing, and leveraging relevant information to construct a well-informed sense of self. Normative identity processing accentuates adherence to mainstream social norms and values, as well as the expectations of significant others. In stark contrast, diffuse–avoidant identity processing refers to an innate and maladaptive tendency to evade or postpone committing to a specific identity. Research has documented that these three identity styles play a crucial role for adolescents in coping with identity conflicts and fostering identity commitments (Phillips & Pittman, 2007). They also influence the process of identity development, which requires a significant variety of psychosocial resources that help to reinforce one’s sense of meaningfulness and facilitate effective regulation of daily life (Adams et al., 2006). It is recognized that the majority of normally behaving individuals, by late adolescence, should be capable of consciously adopting the cognitive strategies reflective of the three identity processing styles (Berzonsky et al., 2007).

**Figure 1:**
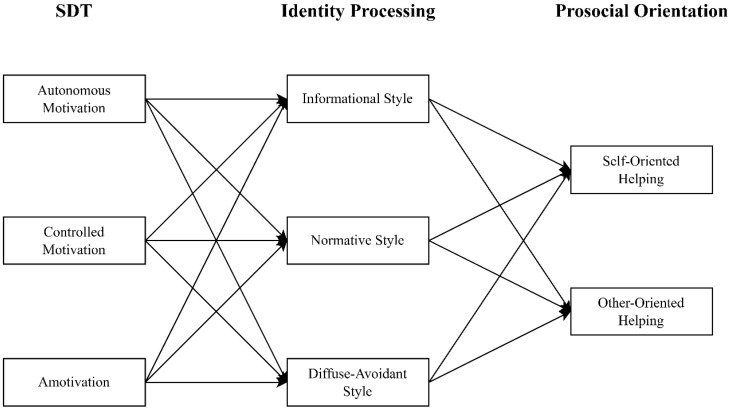
Conceptual research model.

The literature has documented that the three identity processing styles are closely aligned with self-determined motivations and prosocial orientations. Previous research (Soenens et al., 2005a) indicates that individuals with autonomous motivation tend to embrace an informational identity style, as they are primarily driven by internal values and a sense of personal agency. In this self-directed spirit, they are less likely to conform indiscriminately (normative style) or detach fully from identity commitments (diffuse–avoidant style). On the contrary, the controlled motivation, with behaviors being regulated by external approval or internalized pressure is positively associated with a normative identity style, where individuals conform to expectations of significant others or social norms (Smits et al., 2010; Soenens et al., 2005a). Owing to a lack of freedom in self-exploration, controlled motivation is unlikely to be a strong predictor of the informational identity style. Its relationship with the diffuse–avoidant identity style remains uncertain, as controlled motivation may theoretically produce mixed outcomes, i.e., either reinforcing or reducing the propensity to adopt this style. Moreover, those with a tendency towards amotivation, reflecting a lack of direction, purpose, or perceived competence, are expected to exhibit a diffuse–avoidant identity style, characterized by the avoidance of identity-related decisions and procrastination. Unmotivated individuals are also less inclined to subscribe to either the informational style, which requires proactive intrapersonal reflection, or the normative style, which involves commitment to external expectations (Berzonsky, 2008; Koestner et al., 1996). With regard to the links between identity processing style and prosocial orientation, it was found that emerging adults with an informational style are more likely to engage in other-oriented prosocial behaviors, with empathy functioning as a key mediator (Smits et al., 2011). This is because young individuals who opt for the informational style experience increased autonomy in their behavioral choices, which enables greater freedom for identity exploration and nourishes an open-minded and unbiased life attitude (Soenens et al., 2005b). Consequently, the receptive attributes inherent in the informational identity processing style can manifest through voluntary caring for others without unnecessary concerns about external judgment or criticism (Hodgins & Knee, 2002). On the other hand, the normative identity processing style was assumed to be positively related to a high self-oriented helping propensity (Ghag, 2021). In marked contrast to individuals with the informational style, those who adopt the normative style are typified by reserved and defensive attitudes towards the world and society, resulting from a highly controlled and restricted process of identity formation (Soenens et al., 2011). Hence, their behavioral choices hinge heavily on the extent to which their prosocial conducts are able to serve their ego and self-worth, rather than focusing on how others can potentially benefit from their helping (Hodgins & Knee, 2002). Lastly, the diffuse–avoidant identity processing style was observed to have a positive correlation with the self-oriented helping motive (Padilla-Walker et al., 2008). Smits et al. (2011) rationalized this finding by highlighting the social and hedonic values inadvertently sought by diffuse–avoidant individuals, who yearn for recognition to compensate for feelings of emptiness and a lack of purpose in identity development. Accordingly, their helping behaviors are more motivated by self-benefit in the form of social validation than a genuine concern for others. Based on the above review of relevant literature, we formulate the following hypotheses and display our conceptual model in Figure 1:

**H1.**  *Autonomous motivation would be positively associated with the informational identity processing style (1a), and negatively with the normative style (1b) and the diffuse–avoidant style (1c).*

**H2.**  *Controlled motivation would be negatively associated with the informational identity processing style (2a), and positively with the normative style (2b).*

**RQ:**  *How would controlled motivation associate with the diffuse–avoidant identity processing style?*

**H3.**  *Amotivation would be negatively associated with the informational identity processing style (3a) and normative style (3b), and positively with the diffuse–avoidant style (3c).*

**H4.**  *Self-oriented helping would be negatively associated with the informational identity processing style (4a), and positively with the normative style (4b) and the diffuse–avoidant style (4c).*

**H5.**  *Other-oriented helping would be positively associated with the informational identity processing style (5a), and negatively with the normative style (5b) and the diffuse–avoidant style (5c).*

## 3. Method

### 3.1. Participants and Procedure

Participants in this study were recruited with the assistance of a specialized non-profit organization (NPO) that organized several volunteer teaching programs in the summer of 2024. At the end of the spring semester in May 2024 (T1), recruitment information was distributed using email lists and text messages to a random sample of potential volunteers. A total of 386 responses were received from self-identified undergraduate students who expressed strong interest in participating in a chosen volunteer teaching campaign. These students completed an online survey after providing their informed consent, where they reported their demographic information alongside evaluation of self-determined motivation, prosocial orientation, and identity processing style. Among the collected responses, 14 were excluded due to apparent errors (e.g., identical responses across all items) and insufficient response time (i.e., less than 30 s), leaving 372 valid responses. Four months later, in September 2024 (T2), the remaining valid respondents were invited to complete a follow-up survey to re-assess their self-determined motivation and prosocial orientation. Questionnaire used in the study are attached as Supplementary Materials. This time, we collected 322 responses, of which 21 were found to have not actually participated in the said program. 10 invalid responses were removed, and finally, 291 valid responses were prepared for further analysis. Independent *t* tests suggested that attrition of the 81 participants was not related to factors including gender, age, household income, and previous volunteer experience. Among the 291 participants in the final sample, their average age was 18.2 (S.D. = 0.6) and 54% were female; 9% reported having a household income lower than 5999 RMB, 35% had an income between 6000 and 11,999 RMB, 45% had an income between 12,000 and 17,999 RMB, and 11% had an income higher than 18,000 RMB. Lastly, 25% of participants had prior experience in volunteer work.

### 3.2. Measures

#### 3.2.1. Self-Determined Motivations

Volunteer tourists’ autonomous and controlled motivations were evaluated with an adapted version of the Academic Self-Regulation Scale, originally developed by Ryan and Connell (1989). In compliance with previous researchers (De Clerck et al., 2022), we calculated composite scores for autonomous and controlled motivations by averaging the intrinsic motivation and identified regulation subscales for autonomous motivation, and the introjected and external regulation subscales for controlled motivation. On the heels of a prompt “I’m interested in volunteering because…”, eight items (e.g., “I enjoy doing it” and “it is personally important to me”) were used to measure autonomous motivation, and another eight (e.g., “I’m supposed to do so” and “I would feel guilty if I wouldn’t do so”) were used for measuring controlled motivation. Cronbach’s alpha was 0.92 for the autonomous motivation and 0.89 for the controlled motivation scale at T1. The alpha values at T2 were 0.89 and 0.90, respectively. Additionally, volunteers’ amotivation was appraised using four items drawn from the Academic Motivation Scale (Vallerand et al., 1992). The items were slightly modified to cater to the context of volunteer tourism. A sample item was “Honestly, I don’t know; I really feel that I am wasting my time in volunteering”. This subscale had a Cronbach’s alpha of 0.86 at T1 and 0.92 at T2. Participants responded to the items on a 7-point Likert scale, with 1 denoting *not at all like me* and 7 indicating *very much like me*.

#### 3.2.2. Prosocial Orientations

To measure the two dichotomous prosocial orientations, the Self- and Other-Oriented Helping Scale developed and validated by Roth (2008) was utilized. The self-oriented helping subscale, consisting of four items (e.g., “When I’m helping another person, it is important to me that other people will know about that and appreciate me for doing so”), generated a Cronbach’s alpha of 0.86 at T1 and 0.84 at T2. Likewise, the other-oriented helping subscale also comprised four items (e.g., “When I’m helping another person, it is important for me to know how he would like to be helped”) and had a Cronbach’s alpha of 0.86 at T1 and 0.85 at T2. All items were assessed using a 5-point Likert scale, ranging from 1 (*strongly disagree*) to 5 (*strongly agree*).

#### 3.2.3. Identity Processing Styles

The Revised Identity Style Inventory (ISI-5) developed and validated by Berzonsky et al. (2013) was administrated to measure the three distinctive identity processing styles at T1. Prior studies across multiple fields have demonstrated the scale’ reliability and internal consistency (Soenens et al., 2016). Sample items included: “When making important decisions, I like to spend time thinking about my options”, for the 9-item informational style subscale (Cronbach’s alpha = 0.87); “I think it is better to adopt a firm set of beliefs than to be open-minded”, for the 9-item normative style subscale (Cronbach’s alpha = 0.90); and “When personal problems arise, I try to delay acting as long as possible”, for the 9-item diffuse–avoidant style subscale (Cronbach’s alpha = 0.92). Respondents rated each item on a 5-point Likert scale, ranging from 1 (*not at all like me*) to 5 (*very much like me*).

### 3.3. Data Analysis

Structural equation modeling (SEM) was utilized to implement data analysis in this study. It has several unique advantages for managing longitudinal data, particularly in its ability to model complex relationships among latent constructs over time. Different from traditional regression methods, SEM distinguishes between measurement error and structural error, facilitating researchers to model latent variables more accurately by accounting for the unreliability of observed indicators. This contributes to more precise estimates of relationships between theoretical constructs. It also enables the simultaneous testing of multiple dependent variables and time-lagged effects, rendering it ideal for examining causal inferences and developmental trajectories. Furthermore, SEM supports testing of model fit, which allows researchers to assess how well their theoretical model aligns with longitudinal data, thereby providing a robust framework for theory-driven analysis. To conduct SEM analysis, we employed the software R 4.4.0 and its *lavaan* package 0.6-17 to statistically examine our hypothesized model, wherein gender, age, monthly household income, and prior experience in volunteer work were incorporated as control variables on all mediator and outcome variables. Following the practice of Yang et al. (2018), we allowed the error terms of all four T2 outcome variables to covary, as well as those of the three T1 mediators. The acceptance criteria for the model’s goodness of fit were as follows: Comparative Fit Index (CFI) and Tucker–Lewis Index (TLI) greater than 0.95, and Root Mean Square Error of Approximation (RMSEA) less than 0.08.

## 4. Results

Table 1 displays the correlation matrix of the variables under consideration, and the estimated path coefficients appear in Table 2. The model fit was good: *χ*^2^*/df* = 1.13, CFI = 0.98, TLI = 0.98, and RMSEA = 0.02. H1 was set to explore the relationships between autonomous motivation and the three distinctive identity processing styles. Our results indicated that T1 autonomous motivation was positively associated with T1 concurrent informational style (β = 0.68, *p* < 0.001). On the contrary, both T1 normative (β = −0.10, *p* < 0.001) and T1 diffuse–avoidant (β = −0.35, *p* < 0.001) identity styles were negatively predicted by T1 autonomous motivation. With regard to longitudinal correlations, T2 autonomous motivation was related to T1 information style in the positive direction (β = 0.23, *p* < 0.001) and T1 normative style in the negative direction (β = −0.11, *p* < 0.05). It was not significantly associated with T1 diffuse–avoidant style (β = −0.07, *p* = 0.15). Therefore, H1a and H1b were supported. H2 and RQ addressed the connections between controlled motivation and the three identity processing styles. Higher T1 controlled motivation was associated with a lower tendency for T1 informational style (β = −0.10, *p* < 0.01) but a greater inclination of T1 normative style (β = 0.37, *p* < 0.001). T1 controlled motivation had no statistically significant relationship between T1 diffuse–avoidant style (β = 0.02, *p* = 0.76). Regarding longitudinal associations, only T1 normative style, out of the three identity processing styles, was significantly and positively associated with T2 controlled motivation (β = 0.13, *p* < 0.05). H2b was confirmed, whereas the results concerning RQ did not reveal a definitive direction for the relationship between controlled motivation and the diffuse–avoidant identity style. H3 was specified to probe how amotivation relates to identity processing styles. We found that T1 amotivation was negatively associated with T1 informational (β = −0.35, *p* < 0.001) and T1 normative (β = −0.13, *p* < 0.05) styles. However, it had no significant relationship with T1 diffuse–avoidant style (β = −0.06, *p* = 0.32). On longitudinal patterns, all three identity processing styles at T1 were not significantly associated with the likelihood of being not motivated at T2. For H4 (i.e., how self-oriented helping would predict and be predicted by the three identity processing styles), participants’ propensity to engage in self-oriented helping at T1 was positively associated with T1 normative style (β = 0.39, *p* < 0.001) at a significance level, but not significantly with T1 informational (β = −0.02, *p* = 0.60) and T1 diffuse–avoidant (β = 0.05, *p* = 0.50) styles. Moreover, T2 self-oriented helping was positively predicted by T1 normative style (β = 0.30, *p* < 0.001) but negatively by T1 informational (β = −0.16, *p* < 0.001) and T1 diffuse–avoidant (β = −0.12, *p* < 0.01), all at significance levels. As such, only H4b received full support from the longitudinal findings. H5 focused on the relationships between other-oriented helping and different identity processing styles. We did not observe any significant concurrent correlations between these two variables of interest at T1. However, we found that T1 informational style positively predicted T2 other-oriented helping (β = 0.32, *p* < 0.001). Still, the tendency of other-oriented helping at T2 was not significantly related to T1 normative (β = −0.11, *p* = 0.08) and T1 diffuse–avoidant (β = −0.04, *p* = 0.45) styles.

Based upon the above results of direct path coefficients, we further examined two indirect paths of interest. First, the coefficient on the path T1 autonomous motivation → T1 informational style → T2 other-oriented helping was 0.22 (*p* < 0.001). Second, the coefficient on the path T1 controlled motivation → T1 normative style → T2 self-oriented helping was 0.11 (*p* < 0.001). Additionally, we obtained some findings in relation to the control variables. All T1 observations of interest (e.g., T1 autonomous motivation) served as significant and positive predictors of their T2 counterparts (e.g., T2 autonomous motivation). Participants with higher household incomes were more likely to employ the informational style for processing and forming their identity (β = 0.29, *p* < 0.001). Having prior experience in volunteer work also increased their chances of using this identity processing style (β = 0.08, *p* < 0.05). Lastly, participants of an older age exhibited a stronger tendency towards controlled motivation at T2 (β = 0.11, *p* < 0.05).

## 5. Discussion

The current study examined the longitudinal relationships between self-determined motivation and prosocial orientation among volunteer tourists, focusing on the mediating role of identity processing styles. Self-determined motivation pertains to an incentive to act stemming from intrinsic desires, values, or personal satisfaction as opposed to extrinsic pressures or rewards. In our analysis, accordingly, motivations were classified into autonomous, controlled, and amotivation categories. The results revealed that the three categories of motivation had direct associations with volunteers’ concurrent identity processing styles, including informational, normative, and diffuse–avoidant, and indirect associations with their cross-lagged prosocial orientation, which was dichotomized as self-oriented or other-oriented. In the subsequent sections, we first analyze the direct relationships between self-determined motivation and volunteers’ identity processing styles, next evaluate the indirect longitudinal pathways, proceed to discuss the study’s theoretical and practical implications, and finally conclude with an acknowledgement of limitations.

### 5.1. Self-Determined Motivation and Identity Processing Styles

First, volunteer tourists’ autonomous motivation was positively associated with their tendency towards a concurrent informational style, which in turn predicted higher cross-lagged autonomous motivation. Differently, informational style was negatively associated with controlled motivation and amotivation at T1, while their longitudinal relationships were not significant. The informational identity processing style is characterized by proactively seeking and digesting information to achieve a deeper understanding of one’s self and motivations. The finding that volunteer tourists with higher autonomous motivation are more likely to engage in an informational style accentuates the synergy between their intrinsic motivation and self-directed cognitive strategies. Autonomous motivation, driven by personal values and genuine interest, tends to embrace an active approach to self-learning and self-improvement. This aligns naturally with the informational style, in which individuals actively search for, evaluate, and process information to make informed decisions while deepening their understanding of themselves. Such a positive and constructive style not only helps to complement autonomous motivation but also reinforces it by providing greater clarity and a sense of agency (Berzonsky, 2003). Over time, this reciprocally supportive interplay contributes to young volunteer tourists’ personal growth, self-awareness, and long-term engagement in meaningful activities, creating a positive feedback loop that sustains both the behavior and the underlying motivation. On the other hand, the negative concurrent associations of informational style with controlled motivation and amotivation suggest that volunteer tourists who prefer exploration and personal growth are less inclined to conform to external pressures or avoid decision-making. However, the absence of significant longitudinal relationships for these variables in question indicates that the informational style does not necessarily exert an enduring and consistent influence on the strength of controlled motivation and amotivation over time. As such, for promoting sustainable volunteer tourism, it is warranted to cultivate an informational identity processing style to enhance autonomous motivation while recognizing the presence and persistence of maladaptive motivational preferences.

Second, at T1, volunteers’ tendency of normative style was positively associated with their controlled motivation but negatively with their autonomous motivation and amotivation. With respect to longitudinal connections, T1 normative style positively predicted T2 controlled motivation, whereas it negatively predicted T2 autonomous motivation. No significant relationship was found between T1 normative style and T2 amotivation. The normative identity processing style is distinguished by a high degree of conformity to external expectations and societal norms, with limited personal exploration and self-reflection. It is also closely aligned with social support and behavioral control (Smits et al., 2008). The findings related to volunteer tourists’ subscription to the normative style reinforced the notion that those who heavily adhere to external standards and rules tend to have restricted personal agency and autonomy. Their habitual compliance to satisfying external demands or precluding unintended outcomes makes them more prone to controlled motivation, which is galvanized by environmental pressures, rewards, or obligations rather than personal interest or internal desire. Moreover, the dearth of personal exploration inherent in this style results in limited development of intrinsic interests and values. Without sufficient opportunities for introspection and internal decision-making, volunteer tourists are unlikely to develop autonomous motivation, much less personal interests and self-determined goals. Consequently, the normative identity style nourishes controlled motivation and suppresses autonomous motivation, reflecting a stable pattern of behavior that is in obedience with external pressures rather than internal requests. Normative motivation’s failure to predict cross-lagged amotivation may be attributed to its influence being limited to immediate or short-term situations, despite that a strong adherence to external expectations is able to temporarily mute motivation at a given point in time. This seems to suggest that the effects of volunteer tourists’ normative motivation on their amotivation tendency are primarily conditional in lieu of consistent, possibly due to the malleable nature of external expectations and/or the chronic adaptation to new norms over time (Crandall et al., 2002).

Third, we did not observe any significant concurrent relationships between participants’ diffuse–avoidant style and their self-determined motivations. Likewise, we did not detect any longitudinal relationships in this regard. The diffuse–avoidant identity style represents a psychological approach to identity formation in which individuals tend to delay or dodge making decisions relevant to their personal identity. Unlike the other two styles, individuals with this style adopt a procrastinatory stance, often allowing external events or immediate circumstances to dictate their choices. While the diffuse–avoidant identity processing style is typically thought to be less related to autonomous or controlled motivation, it is reasonable to hypothesize it to be positively associated with volunteer tourists’ amotivation tendency. Nevertheless, the results in our study did not confirm either concurrent or longitudinal relationships. This might be ascribed to the style’s innate predisposition towards avoidance and passive decision-making. Volunteer tourists with the diffuse–avoidant style prioritize external circumstances, not in the normative sense but as an escape, over proactive self-reflection, goal-setting, and making commitments. This avoidant inclination may shield them from feelings of disconnection and a lack of fulfillment, which are often accompanied by amotivation, thereby reducing the necessity of exposing to motivational concerns.

### 5.2. Self-Determined Motivation Influencing Prosocial Orientation Mediated Through Identity Processing Styles

In line with previous research, our primary focus lies on two pathways: autonomous motivation with informational identity style and other-oriented helping, and controlled motivation with normative identity style and self-oriented helping. The results reveal that volunteer tourists’ autonomous motivation predicted a greater orientation to help others through its association with higher concurrent informational identity style. This finding echoes the interplay between the nature of volunteering and the development of the self (Wilson, 2000). Volunteering essentially should be an altruistic and prosocial behavior, where autonomous motivation reflects a genuine desire to uphold one’s personal values and enhance the self through providing aid and demonstrating goodwill towards others. At the same time, an informational identity style embodies a proactive approach that involves searching and gleaning relevant information, planning and performing actions, and integrating new insights to develop a coherent sense of self. Individuals with this identity processing style tend to engage in volunteering with curiosity, openness, and a concentration on their personal growth. The informational identity style, therefore, shares an inherent connection with both autonomous motivation and altruistic orientation, playing a pivotal role in shaping and solidifying the identity as a volunteer during its formation or enhancement. It follows that the intrinsic motivation to volunteer not only invites altruistic behavior but also activates a reflective and adaptive process of identity development, illustrating the dynamic reciprocity between self-exploration and prosocial engagement within the context of volunteer tourism.

Whereas the indirect path from T1 autonomous motivation to T2 other-oriented helping through T1 informational identity style was significant, the reverse path—namely, from T1 other-oriented helping to T2 autonomous motivation through the same identity style mediator—was not. Specifically, T1 other-oriented helping did not exhibit a significant association with concurrent informational identity style, despite that the latter produced a positive predictive effect on T2 autonomous motivation. Therefore, this finding fails to confirm the mechanism of a bi-directional relationship. Altruistic orientation, while rooted in self-motivated and heartfelt intentions, appears to function more as an outcome rather than a precursor of autonomous motivation in volunteering. That is, the informational identity style operates independently of immediate altruistic actions, in terms of exerting its influence longitudinally by reinforcing autonomous motivation over time. To cultivate other-oriented interest, individuals require a sufficient input of information that strongly supports self-reflection, exploration, and integration, as in the theorization of the informational identity style. This process entails seeking and processing diverse perspectives, experiences, and feedback to build a deeper understanding of both oneself and the needs of others, which hinges on a well-informed foundation of empathy, values, and purpose.

In relation to another indirect path of interest, we observed that T1 controlled motivation was positively associated with concurrent normative identity style, which predicted the salience of T2 self-oriented helping intention. The positive association between controlled motivation and the concurrent normative identity style at T1 suggests that volunteer tourists incentivized by external pressures or obligations are inclined to comply with socially endorsed norms and values when forming their identity. This reliance on the normative mindset, in turn, contributes to the development of self-oriented helping intentions at T2, indicating that volunteers are likely to approach helping behaviors through a lens of personal benefit or social validation. The further interaction between controlled motivation and normative identity processing can gradually shape prosocial intentions that are self-focused, embodying an alignment between external motivational pressures and identity styles that prioritize conformity and self-interest within social contexts. As volunteer tourists start to internalize these normative frameworks, their helping behaviors become increasingly determined by external expectations and the need for social recognition, rather than by authentic altruism, so as to fulfill personal benefit or the compulsion to conform.

Moreover, our finding suggests a bi-directional relationship among these three variables in question, as there was also a significantly positive indirect association between T1 self-oriented helping and T2 controlled motivation through the mediation of T1 normative identity style. To be precise, volunteers characterized by controlled motivation are more likely to adopt the normative identity style, which predicts self-oriented helping intentions. Those motivated by self-interest to help others similarly resort to the normative identity style, provided conformity to social or organizational norms can afford a sense of psychological safety and comfort, which enhances controlled motivations that are more compatible with external pressures, obligations, or contingencies. Ultimately, this dynamic creates a reinforcing cycle that emphasizes personal need for social validation, wherein volunteer behaviors are propelled less by motivations to benefit others but more by the desire to satisfy external demands. It further highlights that societal norms and expectations can mold not only volunteer identities but also the purpose of prosocial behavior, demonstrating the profound influence of social and environmental cues on volunteer tourists’ personal motivation and moral preferences (Dury et al., 2015).

## 6. Implications

Our findings have several theoretical implications for the literature on volunteer tourism. First, this study delves deeper into the heated discussion on the relationship between volunteers’ motivations and their prosocial orientations (Coghlan & Fennell, 2009; Wearing & McGehee, 2013). Extant research on the motivational dimension of volunteer tourism tends to inspect relatively specific purposes, such as benefiting others, gaining informational and cultural insights, fostering personal and professional development, and promoting socialization (Coghlan & Weiler, 2018). Building on the SDT framework, we classify volunteer tourists’ motivations as intrinsic (autonomous) and extrinsic (controlled) to explore the underlying mechanisms that address why their intention to help others radically differs due to altruistic or egoistic concerns. Furthermore, the relevant literature has predominantly focused on volunteer tourists’ pre-trip motivations (Wearing & McGehee, 2013), which are assumed to be pre-determined and remain static throughout. Our adoption of a longitudinal design recognizes that their motivations may change over time or contingent on different phases of the volunteering experience, in addition to revealing temporally uni- or bi-directional relationships.

Second, our study integrates identity processing styles as a mediator in model specification to connect volunteer tourists’ self-determined motivation and their prosocial orientation. Volunteers’ perception of identity is central to guiding their behavior and steering how they interpret and approach their roles, as well as how they communicate and interact with others. The volunteering experience has the potential to initiate, enhance, or reshape multiple facets of their identity formation as they navigate unprecedented challenges, responsibilities, and social interactions. During this evolutionary journey, different identity processing styles not only impact their motivations and behaviors within the volunteer tourism context but also produce differential influences on their social–cognitive sense of self-concept. It is well documented that identity processing styles closely align with both self-determined motivations (Soenens et al., 2005a) and prosocial orientations (Smits et al., 2011). Additionally, identity development represents a dynamic process that resonates with the longitudinal relationship between these two key variables of interest. As such, it is theoretically reasonable and empirically supported for identity processing styles to serve as the mediator in our investigation.

Third, we observed two prominent and reliable pathways that longitudinally associate the self-determined motivation and prosocial orientation among volunteers. The path from autonomous motivation to other-oriented helping, mediated through the informational style, reinforces the notion that volunteering should be essentially driven by a positive and genuine passion for the cause per se, with participants using a proactive and receptive approach to explore, engage in, and contribute to it. This philanthropic perspective on volunteer tourism, albeit in an extreme or idealized form, corresponds to the principles of positive psychology (Ronel, 2006). Another path from controlled motivation to self-oriented helping through the normative identity style demonstrates that volunteer endeavors can also be incentivized conformity to social or organizational norms, primarily for the sake of fulfilling personal needs for social validation. This appears to be a practical rationale for volunteer behaviors, as true altruism is a rare and valued trait compared to the more widespread occurrence of volunteer activities. Hence, these two pathways epitomize opposing philosophies, including idealistic, pragmatic, or a blend of both, to elucidate volunteer campaigns.

## 7. Limitations and Future Research

Several limitations in this study should be noted and addressed prior to generalizing our findings. First, our study specifically examined the context of volunteer teaching, which may differ drastically from other forms of volunteer tourism (e.g., conservation volunteering, healthcare volunteering), with respect to the primary motivations of participants, the nature of their involvement, and the long-term outcomes for both the volunteers and the communities they visit and serve. Second, college freshmen and sophomores were purposefully chosen as our target sample considering the important developmental transition in their identity development. Although our study adopts a longitudinal design and explores bi-directional relationships, additional time points and extended data collection would make it possible for growth curve modeling to illustrate a more detailed depiction of the dynamics of motivation, identity formation, and prosocial orientation over time. Third, the model currently incorporates only three sets of focal variables, yet future research should explore the inclusion of additional variables and alternative design approaches to provide a more comprehensive understanding. For instance, considering personality factors could generate valuable insights into how individual differences influence the relationships between the key variables. Last but not least, less attention was given to amotivation and the diffuse–avoidant style, probably because volunteering is typically viewed as a form of active participation rather than passive involvement. However, there exist examples where young volunteers are truly unwilling to engage due to a lack of interest or knowledge in the field, yet are pressured by their parents to participate for self-interested reasons, such as enhancing their CV for college admissions. In such cases, amotivation and the diffuse–avoidant style are particularly relevant and call for further consideration.

## Figures and Tables

**Table 1:** Correlation matrix.

	2	3	4	5	6	7	8	9	10	11	12	13
1. aut1	−0.11	−0.10	0.60 ***	−0.04	−0.09	0.70 ***	−0.23 ***	−0.07	−0.15 *	0.01	−0.29 ***	0.18 **
2. con1		−0.04	−0.14 *	0.43 ***	−0.05	−0.14	0.41 ***	0.05	0.14 *	−0.11	0.14 *	−0.12 *
3. amo1			−0.17 **	−0.04	0.82 ***	−0.36 ***	−0.09	−0.05	0.04	0.08	0.02	−0.04
4. aut2				−0.20 ***	−0.21 ***	0.53 ***	−0.23 ***	−0.11	−0.18 **	−0.05	−0.24 ***	0.09
5. con2					−0.05	−0.12 *	0.27 ***	−0.02	0.21 ***	−0.09	0.25 ***	−0.06
6. amo2						−0.28 ***	−0.07	−0.04	0.08	0.07	0.03	0.01
7. inf							−0.17 **	−0.05	−0.11	0.00	−0.25 ***	0.35 ***
8. nor								0.01	0.40 ***	−0.12 *	0.53 ***	−0.18 **
9. dif									0.06	−0.05	−0.06	−0.05
10. sel1										−0.06	0.66 ***	−0.06
11. oth1											−0.09	0.19 **
12. sel2												−0.16 **
13. oth2												

Note. * *p* < 0.05, ** *p* < 0.01, *** *p* < 0.001.

**Table 2:** Estimated coefficients on direct paths.

Direct Path	β	95% ci.lower	95% ci.upper
T1 autonomous → T1 informational	0.68 ***	0.64	0.73
T1 controlled →T1 informational	−0.10 ***	−0.17	−0.03
T1 amotivation → T1 informational	−0.35 ***	−0.42	−0.28
T1 self-oriented → T1 informational	−0.02	−0.09	0.05
T1 other-oriented → T1 informational	0.00	−0.07	0.08
T1 autonomous → T1 normative	−0.16 ***	−0.26	−0.06
T1 controlled → T1 normative	0.37 ***	0.28	0.46
T1 amotivation → T1 normative	−0.13 *	−0.23	−0.02
T1 self-oriented → T1 normative	0.39 ***	0.30	0.49
T1 other-oriented → T1 normative	−0.06	−0.18	0.05
T1 autonomous → T1 diffuse–avoidant	−0.08	−0.19	0.04
T1 controlled →T1 diffuse–avoidant	0.02	−0.10	0.13
T1 amotivation → T1 diffuse–avoidant	−0.06	−0.18	0.06
T1 self-oriented → T1 diffuse–avoidant	0.05	−0.09	0.18
T1 other-oriented → T1 diffuse–avoidant	−0.05	−0.17	0.08
T1 informational → T2 self-oriented	−0.16 ***	−0.24	−0.07
T1 normative → T2 self-oriented	0.30 ***	0.20	0.40
T1 diffuse–avoidant → T2 self-oriented	−0.12 *	−0.21	−0.03
T1 informational → T2 other-oriented	0.32 ***	0.20	0.44
T1 normative → T2 other -oriented	−0.11	−0.23	0.01
T1 diffuse–avoidant → T2 other -oriented	−0.04	−0.15	0.07
T1 informational → T2 autonomous	0.23 ***	0.10	0.37
T1 normative → T2 autonomous	−0.11 *	−0.22	−0.01
T1 diffuse–avoidant → T2 autonomous	−0.07	−0.16	0.02
T1 informational → T2 controlled	−0.04	−0.15	0.07
T1 normative → T2 controlled	0.13 *	0.02	0.24
T1 diffuse–avoidant → T2 controlled	−0.05	−0.15	0.06
T1 informational → T2 amotivation	0.07	−0.01	0.15
T1 normative → T2 amotivation	0.03	−0.05	0.11
T1 diffuse–avoidant → T2 amotivation	0.01	−0.06	0.09

Note. Standardized coefficients are reported. * *p* < 0.05, *** *p* < 0.001.

## Data Availability

The raw data supporting the conclusions of this article will be made available by the authors on request.

## Supplementary Materials

The following supporting information can be downloaded at: https://www.mdpi.com/article/10.3390/bs15050588/s1, S1: Autonomous motivation questionnaire; S2: Controlled motivation questionnaire; S3: Amotivation questionnaire; P1: Self-oriented helping questionnaire; P2: Other-oriented helping questionnaire; I1: Informational style questionnaire; I2: Normative style questionnaire; I3: Diffuse–avoidant style questionnaire.
