# Supplementary material for: A Longitudinal Analysis of the Relationship Between Self-Determined Motivation and Prosocial Orientation of Volunteer Tourists: The Mediating Role of Identity Processing Styles in Young Adults
Source: Behav Sci (Basel). 2025 Apr 27;15(5):588. doi: 10.3390/bs15050588 (PMC12109236; doi:10.3390/bs15050588)
Supplement: Supplementary file 1 [file behavsci-15-00588-s001.zip › behavsci-3513340-supplementary.pdf]

Supplementary Materials

S1. Autonomous motivation

Please evaluate your autonomous motivation on your volunteer activities.							
AM1	I am highly interested in doing this.						
	1<br>Not at all like me	2<br>Slightly like me	3<br>Somewhat like me	4<br>Moderately like me	5<br>Fairly like me	6<br>Quite like me	7<br>Very much like me
AM2	I enjoy doing it.						
	1<br>Not at all like me	2<br>Slightly like me	3<br>Somewhat like me	4<br>Moderately like me	5<br>Fairly like me	6<br>Quite like me	7<br>Very much like me
AM3	It’s fun.						
	1<br>Not at all like me	2<br>Slightly like me	3<br>Somewhat like me	4<br>Moderately like me	5<br>Fairly like me	6<br>Quite like me	7<br>Very much like me
AM4	It’s an exciting thing to do.						
	1<br>Not at all like me	2<br>Slightly like me	3<br>Somewhat like me	4<br>Moderately like me	5<br>Fairly like me	6<br>Quite like me	7<br>Very much like me
AM5	I want to learn new things.						
	1<br>Not at all like me	2<br>Slightly like me	3<br>Somewhat like me	4<br>Moderately like me	5<br>Fairly like me	6<br>Quite like me	7<br>Very much like me
AM6	It is personally important to me.						
	1<br>Not at all like me	2<br>Slightly like me	3<br>Somewhat like me	4<br>Moderately like me	5<br>Fairly like me	6<br>Quite like me	7<br>Very much like me
AM7	This represents a meaningful choice to me.						
	1<br>Not at all like me	2<br>Slightly like me	3<br>Somewhat like me	4<br>Moderately like me	5<br>Fairly like me	6<br>Quite like me	7<br>Very much like me
AM8	This is an important life goal to me.						
	1<br>Not at all like me	2<br>Slightly like me	3<br>Somewhat like me	4<br>Moderately like me	5<br>Fairly like me	6<br>Quite like me	7<br>Very much like me

S2. Controlled motivation

Please evaluate your controlled motivation on your volunteer activities.							
CM1	I’m supposed to do so.						
	1<br>Not at all like me	2<br>Slightly like me	3<br>Somewhat like me	4<br>Moderately like me	5<br>Fairly like me	6<br>Quite like me	7<br>Very much like me
CM2	That’s something others (parents, friends, etc.) force me to do.						
	1<br>Not at all like me	2<br>Slightly like me	3<br>Somewhat like me	4<br>Moderately like me	5<br>Fairly like me	6<br>Quite like me	7<br>Very much like me
CM3	Others (parents, friends, etc.) oblige me to do so.						
	1<br>Not at all like me	2<br>Slightly like me	3<br>Somewhat like me	4<br>Moderately like me	5<br>Fairly like me	6<br>Quite like me	7<br>Very much like me

CM4	That’s what others (e.g., parents, friends) expect me to do.					
	1<br>Not at all like me	2<br>Slightly like me	3<br>Somewhat like me	4<br>Moderately like me	5<br>Fairly like me	6<br>Quite like me	7<br>Very much like me
CM5	I want others to think I’m smart.						
	1<br>Not at all like me	2<br>Slightly like me	3<br>Somewhat like me	4<br>Moderately like me	5<br>Fairly like me	6<br>Quite like me	7<br>Very much like me
CM6	I would feel guilty if I didn’t volunteer.						
	1<br>Not at all like me	2<br>Slightly like me	3<br>Somewhat like me	4<br>Moderately like me	5<br>Fairly like me	6<br>Quite like me	7<br>Very much like me
CM7	I would feel ashamed if I didn’t volunteer.						
	1<br>Not at all like me	2<br>Slightly like me	3<br>Somewhat like me	4<br>Moderately like me	5<br>Fairly like me	6<br>Quite like me	7<br>Very much like me
CM8	I want others to think I’m a good volunteer.						
	1<br>Not at all like me	2<br>Slightly like me	3<br>Somewhat like me	4<br>Moderately like me	5<br>Fairly like me	6<br>Quite like me	7<br>Very much like me

S3. Amotivation

Please evaluate your amotivation on your volunteer activities.							
AMO1	Honestly, I don't know; I really feel that I am wasting my time in volunteering.						
	1<br>Not at all like me	2<br>Slightly like me	3<br>Somewhat like me	4<br>Moderately like me	5<br>Fairly like me	6<br>Quite like me	7<br>Very much like me
AMO2	I once had good reasons for going to volunteer; however, now I wonder whether I should continue.						
	1<br>Not at all like me	2<br>Slightly like me	3<br>Somewhat like me	4<br>Moderately like me	5<br>Fairly like me	6<br>Quite like me	7<br>Very much like me
AMO3	I can't see why I go to volunteer and frankly, I couldn't care less.						
	1<br>Not at all like me	2<br>Slightly like me	3<br>Somewhat like me	4<br>Moderately like me	5<br>Fairly like me	6<br>Quite like me	7<br>Very much like me
AMO4	I don't know; I can't understand what I am doing in volunteering.						
	1<br>Not at all like me	2<br>Slightly like me	3<br>Somewhat like me	4<br>Moderately like me	5<br>Fairly like me	6<br>Quite like me	7<br>Very much like me

P1. Self-oriented helping

Please evaluate your self-oriented helping on your volunteer activities.					
SH1	When I am helping another person, I boast about it.				
	1<br>Strongly disagree	2<br>Disagree	3<br>Neutral	4<br>Agree	5<br>Strongly disagree
SH2	When I’m helping another person, it is important to me that other people will know about that and appreciate me for doing so.				
	1<br>Strongly disagree	2<br>Disagree	3<br>Neutral	4<br>Agree	5<br>Strongly disagree

SH3	When I am helping another person, it is important for me to know that he/she appreciates me for doing so.				
	1<br>Strongly disagree	2<br>Disagree	3<br>Neutral	4<br>Agree	5<br>Strongly disagree
SH4	I only help someone else if others know about it.				
	1<br>Strongly disagree	2<br>Disagree	3<br>Neutral	4<br>Agree	5<br>Strongly disagree

P2. Other-oriented helping

Please evaluate your other-oriented helping on your volunteer activities.					
OH1	When I'm helping another person, it is important for me to know how he would like to be helped.				
	1<br>Strongly disagree	2<br>Disagree	3<br>Neutral	4<br>Agree	5<br>Strongly disagree
OH2	When I help someone else, I try to be attentive to his or her needs.				
	1<br>Strongly disagree	2<br>Disagree	3<br>Neutral	4<br>Agree	5<br>Strongly disagree
OH3	When I don't succeed at helping others, I think about how I can help in a more effective way next time.				
	1<br>Strongly disagree	2<br>Disagree	3<br>Neutral	4<br>Agree	5<br>Strongly disagree
OH4	If someone refuses my help, I try to understand why.				
	1<br>Strongly disagree	2<br>Disagree	3<br>Neutral	4<br>Agree	5<br>Strongly disagree

I1. Informational style

Please evaluate your informational style on your volunteer activities.					
IS1	When making important decisions, I like to spend time thinking about my options.				
	1<br>Not at all like me	2<br>Slightly like me	3<br>Moderately like me	4<br>Quite like me	5<br>Very much like me
IS2	When facing a life decision, I take into account different points of view before making a choice.				
	1<br>Not at all like me	2<br>Slightly like me	3<br>Moderately like me	4<br>Quite like me	5<br>Very much like me
IS3	It is important for me to obtain and evaluate information from a variety of sources before I make important life decisions.				
	1<br>Not at all like me	2<br>Slightly like me	3<br>Moderately like me	4<br>Quite like me	5<br>Very much like me
IS4	When making important decisions, I like to have as much information as possible.				
	1<br>Not at all like me	2<br>Slightly like me	3<br>Moderately like me	4<br>Quite like me	5<br>Very much like me
IS5	When facing a life decision, I try to analyze the situation in order to understand it.				
	1<br>Not at all like me	2<br>Slightly like me	3<br>Moderately like me	4<br>Quite like me	5<br>Very much like me
IS6	Talking to others helps me explore my personal beliefs.				
	1<br>Not at all like me	2<br>Slightly like me	3<br>Moderately like me	4<br>Quite like me	5<br>Very much like me

IS7	I handle problems in my life by actively reflecting on them.				
	1<br>Not at all like me	2<br>Slightly like me	3<br>Moderately like me	4<br>Quite like me	5<br>Very much like me
IS8	I periodically think about and examine the logical consistency between my values and life goals.				
	1<br>Not at all like me	2<br>Slightly like me	3<br>Moderately like me	4<br>Quite like me	5<br>Very much like me
IS9	I spend a lot of time reading or talking to others trying to develop a set of values that makes sense to me.				
	1<br>Not at all like me	2<br>Slightly like me	3<br>Moderately like me	4<br>Quite like me	5<br>Very much like me

I2. Normative style

Please evaluate your normative style on your volunteer activities.					
NS1	I automatically adopt and follow the values I was brought up with.				
	1<br>Not at all like me	2<br>Slightly like me	3<br>Moderately like me	4<br>Quite like me	5<br>Very much like me
NS2	I think it is better to adopt a firm set of beliefs than to be open-minded.				
	1<br>Not at all like me	2<br>Slightly like me	3<br>Moderately like me	4<br>Quite like me	5<br>Very much like me
NS3	I think it’s better to hold on to fixed values rather than to consider alternative value systems.				
	1<br>Not at all like me	2<br>Slightly like me	3<br>Moderately like me	4<br>Quite like me	5<br>Very much like me
NS4	When I make a decision about my future, I automatically follow what close friends or relatives expect from me.				
	1<br>Not at all like me	2<br>Slightly like me	3<br>Moderately like me	4<br>Quite like me	5<br>Very much like me
NS5	I prefer to deal with situations in which I can rely on social norms and standards.				
	1<br>Not at all like me	2<br>Slightly like me	3<br>Moderately like me	4<br>Quite like me	5<br>Very much like me
NS6	I have always known what I believe and don’t believe; I never really have doubts about my beliefs.				
	1<br>Not at all like me	2<br>Slightly like me	3<br>Moderately like me	4<br>Quite like me	5<br>Very much like me
NS7	I never question what I want to do with my life because I tend to follow what important people expect me to do.				
	1<br>Not at all like me	2<br>Slightly like me	3<br>Moderately like me	4<br>Quite like me	5<br>Very much like me
NS8	When others say something that challenges my personal values or beliefs, I automatically disregard what they have to say.				
	1<br>Not at all like me	2<br>Slightly like me	3<br>Moderately like me	4<br>Quite like me	5<br>Very much like me
NS9	I strive to achieve the goals that my family and friends hold for me.				
	1<br>Not at all like me	2<br>Slightly like me	3<br>Moderately like me	4<br>Quite like me	5<br>Very much like me

I3. Diffuse-avoidant style

Please evaluate your diffuse-avoidant style on your volunteer activities.					
DS1	When personal problems arise, I try to delay acting as long as possible.				
	1<br>Not at all like me	2<br>Slightly like me	3<br>Moderately like me	4<br>Quite like me	5<br>Very much like me
DS2	I’m not sure where I’m heading in my life; I guess things will work themselves out.				
	1<br>Not at all like me	2<br>Slightly like me	3<br>Moderately like me	4<br>Quite like me	5<br>Very much like me
DS3	My life plans tend to change whenever I talk to different people.				
	1<br>Not at all like me	2<br>Slightly like me	3<br>Moderately like me	4<br>Quite like me	5<br>Very much like me
DS4	Who I am changes from situation to situation.				
	1<br>Not at all like me	2<br>Slightly like me	3<br>Moderately like me	4<br>Quite like me	5<br>Very much like me
DS5	I try not to think about or deal with problems as long as I can.				
	1<br>Not at all like me	2<br>Slightly like me	3<br>Moderately like me	4<br>Quite like me	5<br>Very much like me
DS6	I try to avoid personal situations that require me to think a lot and deal with them on my own.				
	1<br>Not at all like me	2<br>Slightly like me	3<br>Moderately like me	4<br>Quite like me	5<br>Very much like me
DS7	When I have to make a decision, I try to wait as long as possible in order to see what will happen.				
	1<br>Not at all like me	2<br>Slightly like me	3<br>Moderately like me	4<br>Quite like me	5<br>Very much like me
DS8	It doesn’t pay to worry about values in advance; I decide things as they happen.				
	1<br>Not at all like me	2<br>Slightly like me	3<br>Moderately like me	4<br>Quite like me	5<br>Very much like me
DS9	I am not really thinking about my future now, it is still a long way off.				
	1<br>Not at all like me	2<br>Slightly like me	3<br>Moderately like me	4<br>Quite like me	5<br>Very much like me
